# Bilateral Internal Carotid Artery Occlusion, an Unusual Clinical Entity in a Young Adult

**DOI:** 10.7759/cureus.15971

**Published:** 2021-06-27

**Authors:** Monika Karki, Pradeep Kumar Devarakonda, Lohitha Dhulipalla, Meghana Pattipati, Cesar Ayala-Rodriguez

**Affiliations:** 1 Internal Medicine, The Brooklyn Hospital Center, Brooklyn, USA; 2 Cardiology, The Brooklyn Hospital Center - Mount Sinai Heart, Brooklyn, USA

**Keywords:** internal carotid artery (ica), bilateral internal carotid artery stenosis, : ischemic stroke, hypoesthesia, carotid endarterectomy (cea)

## Abstract

Bilateral internal carotid artery occlusion (BICAO) is a rare disease leading to serious cerebrovascular disease and complications including recurrent ischemic stroke or death. There are very few cases reported on BICAO, especially among young adults. The clinical presentation can range from asymptomatic to fatal ischemic stroke depending upon the presence of adequate collateral blood flow. Here we report a case of BICAO in a 31-year-old female who presented with intermittent left-sided hemiparesis for one day and was subsequently found to have complete occlusion of the bilateral intracranial internal carotid arteries on CT angiography (CTA). Magnetic resonance imaging (MRI) brain showed sub-acute right middle cerebral artery (MCA), acute left middle cerebral artery, and anterior cerebral artery infarcts (ACA). Being outside the window for thrombolysis, she was successfully treated with balloon angioplasty of the left internal carotid artery (ICA) and started on dual antiplatelet therapy. The case illustrates the importance of evaluating for cerebrovascular disease when someone presents with stroke-like symptoms even in the young adult population, as prompt treatment can be lifesaving.

## Introduction

Stroke is a common neurological disease and accounts for the second leading cause of death in the world [1] and a fourth leading cause of death in the United States [2]. The prevalence of stroke in the U.S. is about 3% accounting for around 7 million people [2]. Risk factors for stroke are categorized as modifiable and nonmodifiable. Modifiable risk factors are further classified into medical risk factors such as hypertension, carotid stenosis, atrial fibrillation, and lifestyle risk factors such as smoking, dietary habits, physical inactivity [2].

The precise incidence rates of internal carotid artery (ICA) occlusion are unknown as many people remain asymptomatic however, in one of the retrospective studies among symptomatic patients, it was reported as 6/100,000 population [3]. Unilateral ICA occlusion accounts for the majority of the stroke patients accounting for about 99.6% while bilateral ICA occlusion accounts for 0.4% of the stroke patients [1].

## Case presentation

**Figure 1:**
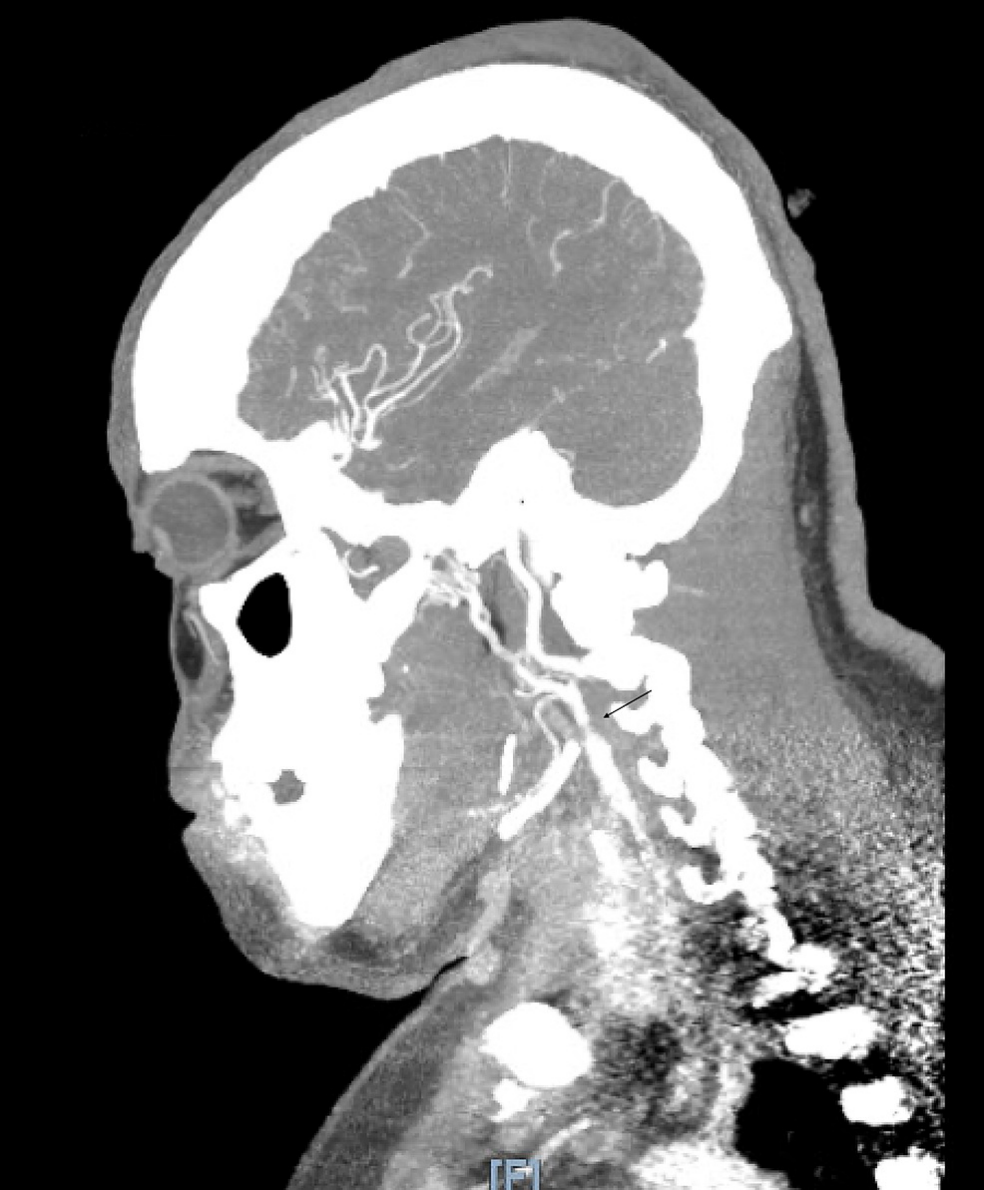
A sagittal image of CTA head and neck, arrow showing stenosis in proximal ICA CTA: CT angiography; ICA: Internal carotid artery.

**Figure 2:**
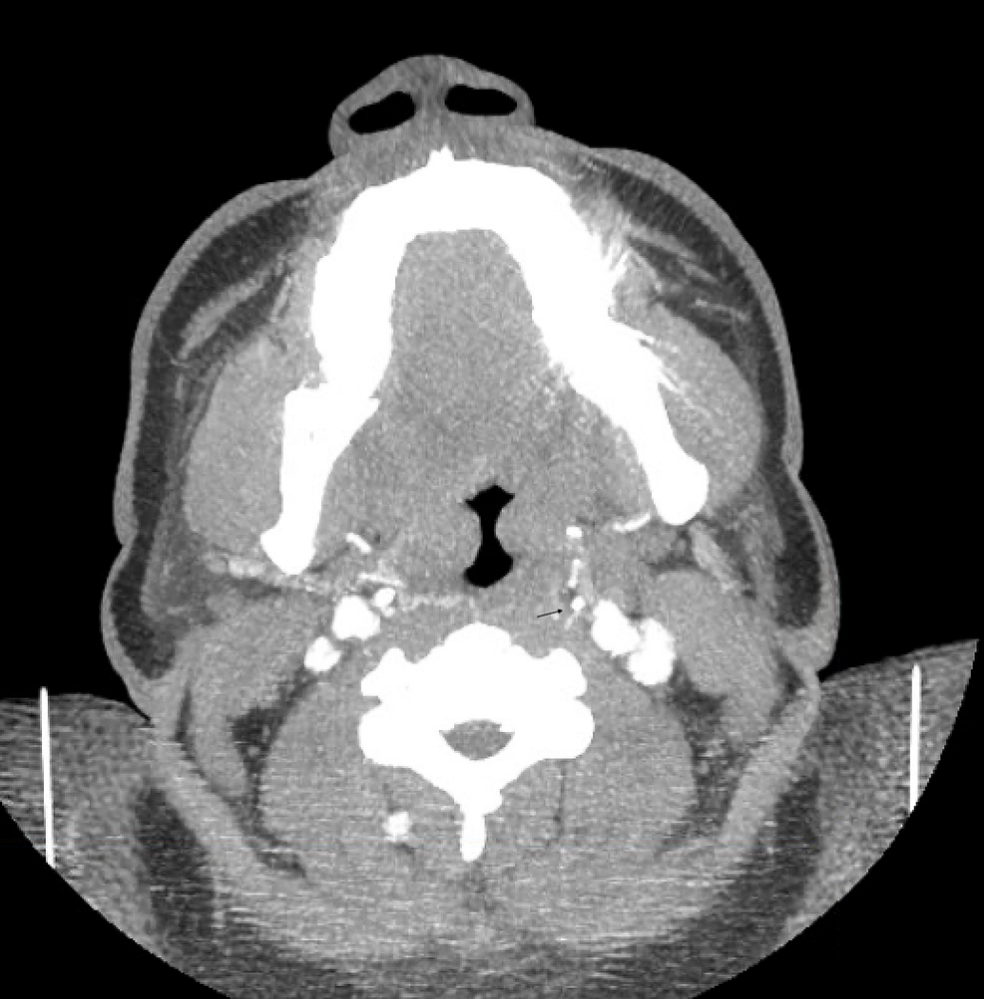
Axial image, stenosis is shown with arrow

**Figure 3:**
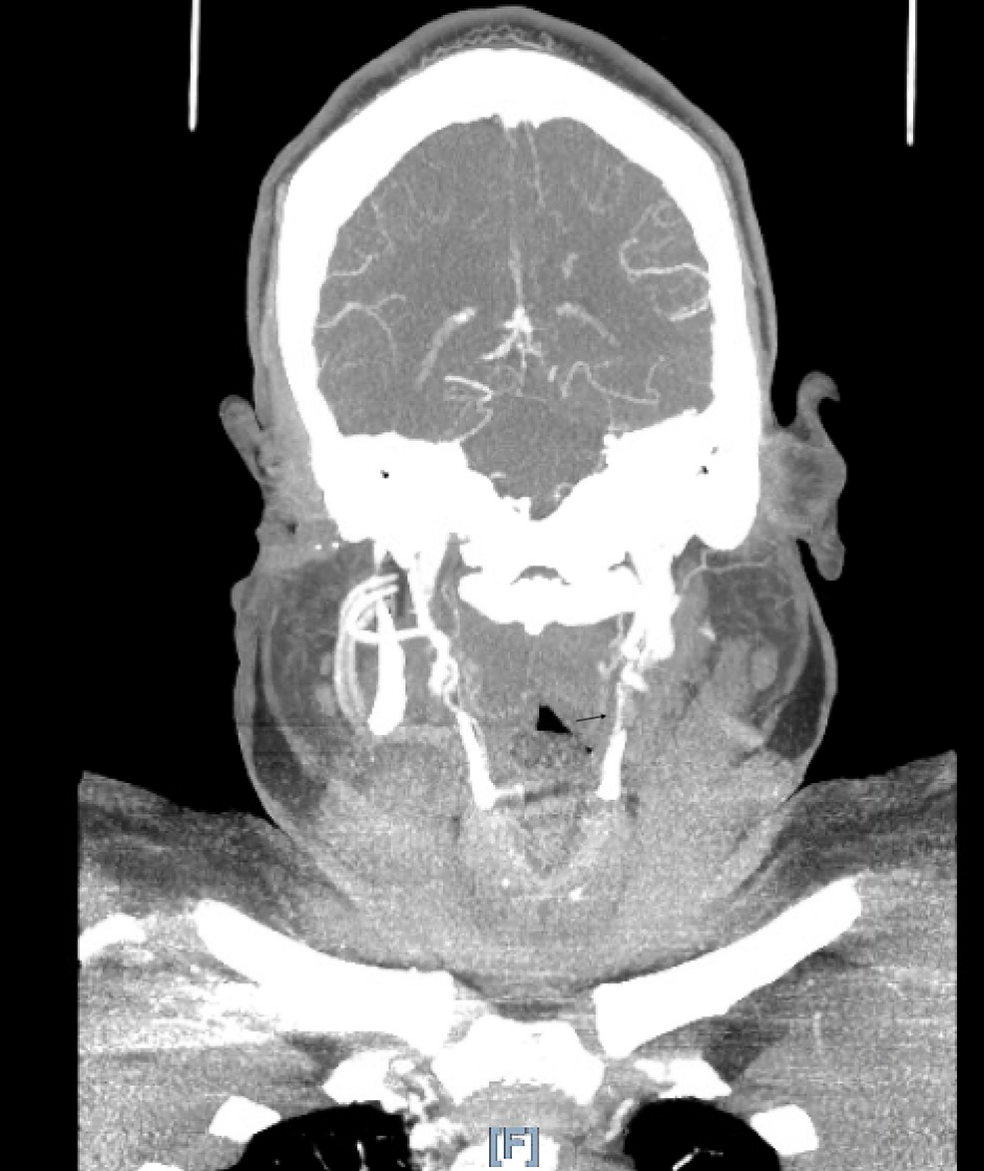
Coronal image, stenosis is shown with arrow

**Figure 4:**
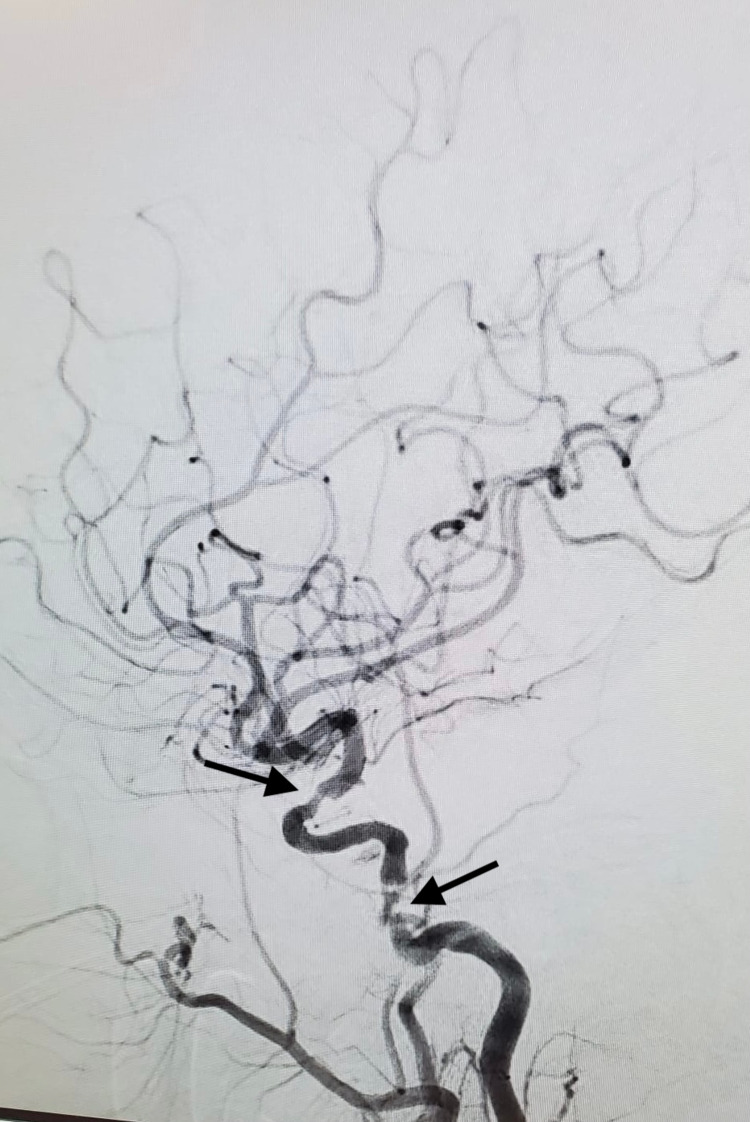
Cerebral angiogram, stenosis of left ICA at multiple level shown with arrow ICA: Internal carotid artery

**Figure 5:**
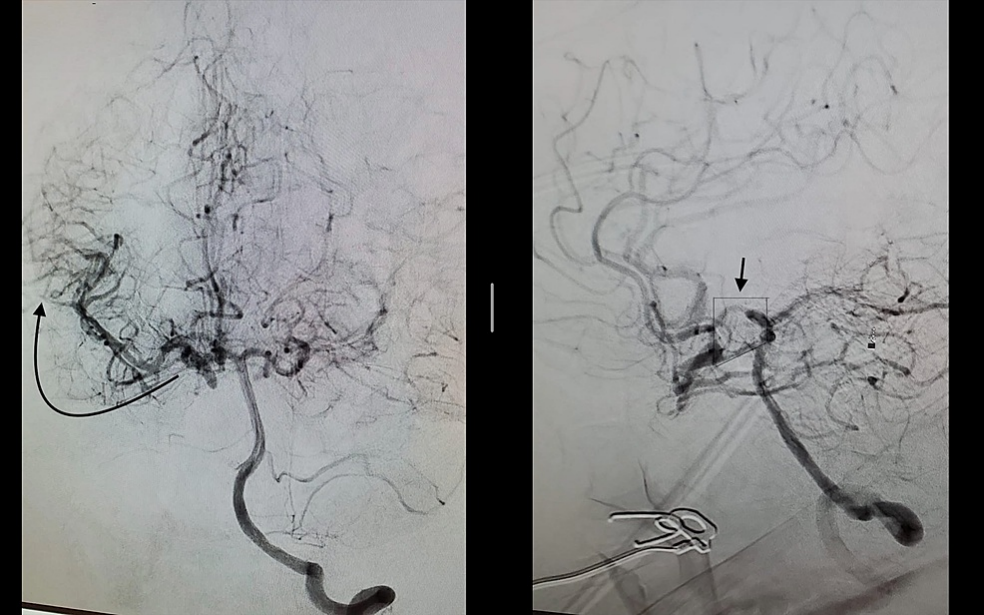
Cerebral angiogram, right MCA (curved arrow in left picture) supplied retrogradely via PComm (small arrow in right picture) MCA: Middle cerebral artery; PComm: Posterior communicating arteries.

A 31-year-old female with a past medical history of diabetes mellitus, hypertension, and morbid obesity presented to our hospital with intermittent right-sided body weakness and numbness for one day. Her initial symptoms onset one day before her presentation, associated with hypoesthesia followed by hemiparesis of her right face, right upper extremity (UE), and right lower extremity (LE) lasting for 30 seconds, which was followed by intermittent right LE paresis. She reported no dysphagia, dysarthria, or visual symptoms, and had no prior history of a similar episode. Her initial National Institute of Health Stroke Scale and Score (NIHSS) was 2 for mild weakness in the right lower extremities. Comprehensive laboratory evaluations were remarkable for a hemoglobin A1C of 15%, an elevated erythrocyte sedimentation rate greater than 100mm/hr, a C-reactive protein of 12mg/L, and serum triglycerides of 240mg/dL. A non-contrast CT of the head showed no evidence of hemorrhage, ischemic changes, or structural abnormalities, while CT angiography of the head and neck showed complete occlusion of her bilateral intracranial internal carotid arteries (ICA), complete right cervical ICA stenosis, and severe multifocal left ICA stenosis (Figures 1-4). MRI brain showed subacute infarcts in the right middle cerebral artery (MCA) territory, with acute infarcts in the left MCA and anterior cerebral artery (ACA) territories. She then underwent a diagnostic cerebral angiogram, which again demonstrated complete occlusion of the bilateral ICA. Her right anterior and middle cerebral arteries were found to be perfused by collateral flow from the posterior communicating arteries (PComm) (Figure 5). She was successfully treated with balloon angioplasty to the left internal carotid artery with significant improvement in stenosis. The patient was then started on high-intensity statin, dual antiplatelet therapy with aspirin and clopidogrel for 90 days followed by life-long aspirin and statin. Upon discharge, blood pressure was well controlled with lisinopril 10mg, and follow-up appointments were made for vascular surgery, neurology, and rheumatology for further autoimmune/hyper-coagulable/vasculitis workup and management outpatient.

## Discussion

Stroke is the main leading cause for adult disability in the USA [2]. There are very few cases reported on bilateral internal carotid artery occlusion (BICAO) and this condition is likely even rarer among young adults.

We presented this case of a young patient with bi-hemispheric stroke with findings of BICAO of unknown etiology. Her history was notable for risk factors for atherosclerotic disease including diabetes mellitus (DM) and hypertension (HTN). The differential etiology for BICAO, especially among young adults, would include cardioembolic phenomena, fibromuscular dysplasia (FMD), large-vessel vasculitis, early atherosclerosis, congenital abnormalities as Moyamoya disease, and hypercoagulable states such as anti-phospholipid antibody syndrome.

Cardioembolic sources were excluded in this case based on a transthoracic echocardiography (TTE) assessment. Large-vessel vasculitis was felt to be unlikely for this patient given the absence of correlating symptoms like headache, jaw claudication, visual disturbances, or loss of peripheral pulses, along with her normal thoracic and abdominal aorta as seen in CT angiography of the chest, abdomen, and pelvis. Congenital abnormalities as Moyamoya disease were ruled out as stenosis was of proximal ICA rather than distal ICA or vessels of the circle of Willis [4]. A hypercoagulable condition could not be ruled out despite this patient had no history of any venous thrombosis or history of abortions, since MRI venous was not performed nor the complete autoimmune workup was completed as the patient missed several outpatients follow-up appointments. Anti-cardiolipin antibody was never tested in this patient, however, a screen for anti-nuclear antibodies was negative. Fibromuscular disease was less likely as her angiogram did not show the hallmark “string-of-beads” appearance associated with this disease and her CT of the abdomen and pelvis showed normal celiac, superior mesenteric artery (SMA), inferior mesenteric artery (IMA), and bilateral renal arteries. Ultimately, without a definitive underlying etiology, the patient was treated for atherosclerotic disease given the risk factors from her history.

Brightness modulation (B Mode) ultrasonography can act as a useful diagnostic study for differentiating normal and severely stenotic vessels. Carotid duplex ultrasonography is considered among one of the highest sensitive and specific studies to diagnose total carotid occlusion. MRA with gadolinium and CT angiography (CTA) are considered as sensitive as ultrasonography in diagnosing internal carotid artery occlusion, while the gold standard imaging modality is digital subtraction angiography, which is an invasive test usually only required when MRA and ultrasonography are unable to differentiate near or complete occlusion [5].

The definitive treatment for BICAO remains unclear [6]. One approach would include a combination of medical therapy with dual antiplatelet (90 days duration) + statin therapy and surgical revascularization, while another is to pursue medical treatment alone [7]. The surgical options available include vascular bypass and external carotid artery revascularization via carotid endarterectomy (CEA) or carotid artery stenting (CAS). According to a meta-analysis study performed by Mylonas et al., there was no significant difference in the outcome between medical therapy or revascularization treatment in these patients [8]. A study performed by Friedman et al. indicated that neither extracranial-intracranial bypass alone nor medical therapy alone was effective methods to restore sufficient blood flow to improve patient symptoms [9]. Due to the low reported incidence of this condition, definitive data on outcomes is necessarily limited.

## Conclusions

In this care report, we emphasized the importance of timely recognition of stroke-like symptoms, diagnostic options, and treatment options in a young patient found to have bilateral ICA occlusion. Diagnostic options include CTA head and neck to evaluate for artery occlusion, CT head / MRI brain for ischemia. Treatment options include medical management alone, such as dual anti-platelet therapy and thrombolysis, or aggressive medical and surgical management such as carotid endarterectomy, stent placement, or balloon angioplasty.
